# Diagnostic Accuracy of Ultrasonographic Respiratory Variation in the Inferior Vena Cava, Subclavian Vein, Internal Jugular Vein, and Femoral Vein Diameter to Predict Fluid Responsiveness: A Systematic Review and Meta-Analysis

**DOI:** 10.3390/diagnostics12010049

**Published:** 2021-12-27

**Authors:** Do-Wan Kim, Seungwoo Chung, Wu-Seong Kang, Joongsuck Kim

**Affiliations:** 1 Department of Thoracic and Cardiovascular Surgery, Chonnam National University Hospital, Chonnam National University Medical School, Gwangju 61469, Korea; maskjoa@naver.com; 2 Department of Critical Care Medicine, Gyeongsang National University Changwon Hospital, Changwon 51472, Korea; psinizu@hanmail.net; 3 Department of Trauma Surgery, Jeju Regional Trauma Center, Cheju Halla General Hospital, Jeju 63127, Korea; jsknight68@gmail.com

**Keywords:** fluid responsiveness, inferior vena cava, internal jugular vein, subclavian vein, femoral vein, ultrasonography

## Abstract

This systematic review and meta-analysis aimed to investigate the ultrasonographic variation of the diameter of the inferior vena cava (IVC), internal jugular vein (IJV), subclavian vein (SCV), and femoral vein (FV) to predict fluid responsiveness in critically ill patients. Relevant articles were obtained by searching PubMed, EMBASE, and Cochrane databases (articles up to 21 October 2021). The number of true positives, false positives, false negatives, and true negatives for the index test to predict fluid responsiveness was collected. We used a hierarchical summary receiver operating characteristics model and bivariate model for meta-analysis. Finally, 30 studies comprising 1719 patients were included in this review. The ultrasonographic variation of the IVC showed a pooled sensitivity and specificity of 0.75 and 0.83, respectively. The area under the receiver operating characteristics curve was 0.86. In the subgroup analysis, there was no difference between patients on mechanical ventilation and those breathing spontaneously. In terms of the IJV, SCV, and FV, meta-analysis was not conducted due to the limited number of studies. The ultrasonographic measurement of the variation in diameter of the IVC has a favorable diagnostic accuracy for predicting fluid responsiveness in critically ill patients. However, there was insufficient evidence in terms of the IJV, SCV, and FV.

## 1. Introduction

Achieving a satisfactory response to fluid replacement in critically ill patients has remained a challenging issue [1,2,3,4]. An insufficient fluid volume can lead to low cardiac output (CO), which may result in reduced tissue perfusion [1,2]. However, excessive fluid infusion might also be detrimental. As depicted by the Frank–Starling curve, an increase in preload does not correspond to an equal increase in stroke volume (SV) when it reaches the maximum slope and plateau [5]. Excessive fluid volume is a significant risk factor for acute lung injury, bowel edema, and compartment syndrome [6,7]. Therefore, it is crucial to determine whether the patient needs additional fluid or not. However, it is not easy to precisely predict fluid responsiveness before fluid administration because the etiology of shock associated with diverse aspects of fluid balance is difficult to ascertain. This is despite the fact that many clinical manifestations of shock such as low blood pressure, tachycardia, altered mental state, cool clammy skin, or low urine output have been described [8]. To evaluate fluid responsiveness, information about the increase in SV after fluid challenge is useful. Invasive methods used in the past include the Swan–Ganz catheter, which directly measures capillary wedge pressure, and has been the gold standard for CO or SV measurement [9]. However, it is a substantially invasive and difficult procedure, especially in patients with cardiovascular instability [4,9]. Therefore, to overcome the shortcomings of the Swan–Ganz measurement method, a pulse wave analysis method that attempts to measure CO or SV has been proposed [10]. Other minimally invasive and non-invasive methods such as arterial pulse wave analysis also have several limitations in terms of artifact validation, arterial compliance, alteration in vasomotor tone, or non-pulsatile blood flow [10]. Moreover, the above-mentioned techniques are unable to predict fluid responsiveness before the fluid challenge.

Recently, several researchers have applied a point-of-care ultrasound for critically ill patients [11]. Ultrasonography is non-invasive, and its cost is relatively low. It can measure SV effectively [11]. In addition, ultrasonography can detect the variation of IVC diameter (ΔIVC), which reflects the cardiac preload [11,12]. By measuring the cardiac preload, it is possible to predict volume status and fluid responsiveness. The measurement of the IVC diameter is easily performed via a subxiphoid view even by non-highly trained operators, whereas measuring the SV via an echocardiogram requires an experienced intensivist or cardiologist. In addition, the internal jugular vein (IJV), subclavian vein (SCV), and femoral vein (FV) are easier to visualize because they are more superficial than the IVC. The diameter of the IVC varies with inspiration and expiration [12]. In patients who breathe spontaneously, intrathoracic pressure decreases during inspiration; this results in accelerated venous return. During expiration, intrathoracic pressure increases, and venous return decreases [12]. Consequently, the IVC diameter decreases during inspiration and increases during expiration. When mechanical ventilation is employed, this phenomenon reverses. However, the ΔIVC is not always visible in patients with obesity, intraabdominal fluid collection, or bowel gas. Thus, other large veins might be used as alternatives in such patients.

To date, several meta-analyses have demonstrated that ΔIVC showed favorable outcomes [12,13]. However, these evaluated data up to 2017, and many more studies have been published since then. Moreover, there has been no systematic review and meta-analysis regarding other large veins such as the IJV, SCV, or FV. To update the evidence on ΔIVC and explore its alternatives, we conducted a systematic review and meta-analysis for respiratory variation in the diameters of the IVC, IJV, SCV, and FV.

## 2. Materials and Methods

### 2.1. Published Study Search and Selection Criteria

This study was performed according to the Preferred Reporting Items for Systematic Reviews and Meta-Analysis of Diagnostic Test Accuracy (PRISMA-DTA) statement published study search and selection criteria [14]. The preset protocol of this study was registered on PROSPERO (CRD42020206037, https://www.crd.york.ac.uk/prospero/, last accessed date: 21 October 2021). Relevant articles were obtained by searching PubMed, EMBASE, and Cochrane databases through 21 October 2021. These databases were searched using the following keywords: “((subclavian vein) OR (inferior vena cava) OR (internal jugular vein) OR (femoral vein)) AND ((fluid responsiveness) OR volume) AND (diameter OR collapsibility OR measurement) AND (ultrasonography OR ultrasound OR sonography OR sonographic OR (point of care))”. We also manually searched the reference lists of relevant articles. The titles and abstracts of all searched articles were screened for exclusion. Review articles and previous meta-analyses were also screened to obtain additional eligible studies. The search results were then reviewed and articles were included if the study investigated the diagnostic accuracy of the IVC, SCV, IJV, and FV to predict fluid responsiveness.

The inclusion criteria for diagnostic test accuracy (DTA) reviews were as follows: (1) the study population included patients who received fluid replacement due to sepsis, hypovolemia, or circulatory failure; (2) an ultrasonographic measurement of the respiratory variability of the IVC, SCV, IJV, and FV diameter was performed as an index test; (3) tests that enabled measurement of fluid responsiveness were performed for reference standard; (4) the primary outcome of the study was the diagnostic accuracy of ultrasonographic respiratory variability of the diameters of IVC, SCV, IJV, and FV to predict fluid responsiveness; (5) adequate information was provided to build a 2-by-2 contingency table consisting of true positive (TP), false positive (FP), false negative (FN), and true negative (TN) outcomes. Articles that involved another disease, those that did not provide 2-by-2 contingency table information, non-original articles, non-human studies, pediatric studies, or those published in a language other than English were excluded.

### 2.2. Data Extraction

Data from all eligible studies were extracted by two investigators. Extracted data from each of the eligible studies included: the first author’s name, year of publication, study location, study design and period, number of patients analyzed, measured vein, index test, threshold of index test, reference standard, device used for the reference standard, threshold of reference standard, and fluid responsiveness. The number of TP, FP, FN, and TN for the index test in predicting fluid responsiveness were collected. If the eligible study reported multiple thresholds and accuracy of the index test, we extracted the subset with optimal threshold or highest performance.

### 2.3. Quality Assessment

All studies were independently reviewed by two investigators. Disagreements concerning the study selection and data extraction were resolved by consensus. As recommended by the Cochrane Collaboration, the Quality Assessment of Diagnostic Accuracy Studies (QUADAS)-2 tool was used to evaluate the risk of bias in DTA [15]. Disagreements in this regard were resolved by discussion with the third independent author. The QUADAS-2 assesses four domains for bias and applicability as follows: (1) patient selection; (2) index test; (3) reference standard; (4) flow and timing.

### 2.4. Statistical Analysis

For statistical analysis using meta-analysis, we used the “metandi” and “midas” modules of Stata version 17.0 (Stata Corporation, College Station, TX, USA) and “mada” package of the R programming language, version 4.0.3 (R foundation, Vienna, Austria). QUADAS-2 assessment was performed using Review Manager Software 5.4 (The Cochrane Collaboration, Oxford, Copenhagen, Denmark). We constructed a 2-by-2 contingency table (TP, FP, FN, TN) by calculating or extracting from each primary study. For rigorous statistical analysis and heterogeneity across the studies, we used both the hierarchical summary receiver operating characteristics (HSROC) model [16] and the bivariate model [17]. A bivariate mixed-effects regression model for the synthesis of diagnostic test data and the derived logit estimates of sensitivity, specificity, and respective variances was used to construct a hierarchical summary ROC curve [17]. The HSROC model assumes that there is an underlying ROC curve in each study with parameters that characterize the accuracy and asymmetry of the curve [16]. An area under the ROC curve (AUROC) close to 1 and 0.5 indicated a strong test and poor test, respectively. Results with *p*-values < 0.05 were considered statistically significant. To investigate the heterogeneity, I2 was calculated from results as I2 = 100% × (Q − df)/Q, where Q is Cochran’s heterogeneity statistics and df is the degree of freedom [18]. I2 lies between 0% and 100%. A value of 0% indicates no observed heterogeneity and values greater than 50% are considered to indicate substantial heterogeneity. To detect the threshold effect, Spearman’s correlation coefficient between sensitivity and specificity was calculated after logit transformation. The HSROC shape (asymmetry) parameter was β (beta), where β = 0 corresponds to a symmetric ROC curve in which the diagnostic odds ratio does not vary along the curve [16]. Due to the trade-off between sensitivity and specificity, we used bivariate random-effects modeling of sensitivity and specificity as we expected that this pair of performance measures will be interdependent. We used the bivariate box plot that describes the degree of interdependence including the central location and identification of any outliers [19]. The inner oval represents the median distribution while the outer oval represents the 95% confidence bound. The skewness provides indirect evidence of some threshold variability [19]. A multiple univariable bivariate meta-regression was conducted to investigate the possible source of heterogeneity. Covariates were manipulated as mean-centered continuous or dichotomous (yes = 1. No = 0) fixed effects. Publication bias was first assessed visually using a scatter plot. We used the diagnostic log odds ratio (lnDOR), which should have a symmetrical funnel shape when publication bias is absent [20]. Formal testing for publication bias was conducted by the regression of lnDOR against the square root of the effective sample size, with *p* < 0.05 for the slope coefficient indicating significant asymmetry [20].

## 3. Results

### 3.1. Selection and Characteristics

**Figure 1:**
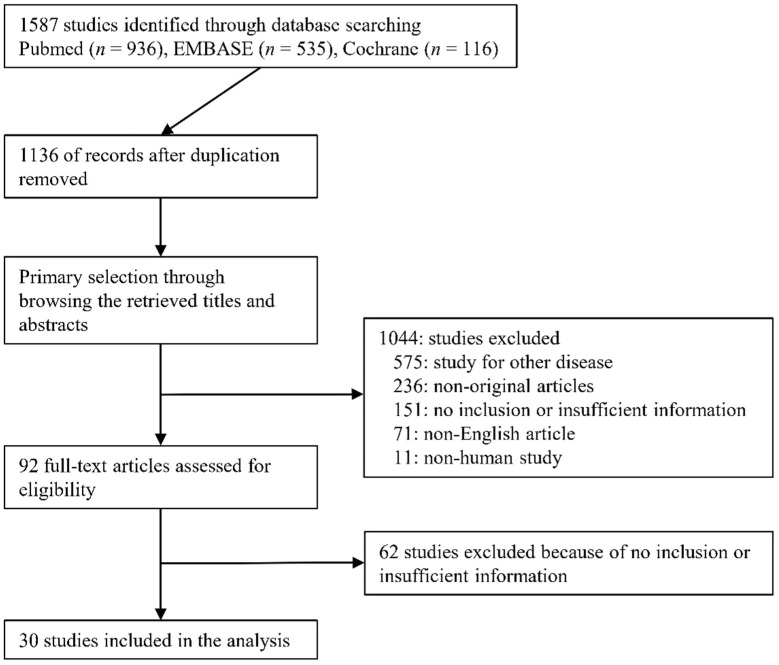
Flow diagram for identification of eligible studies.

A total of 1587 studies were identified through searching databases. After removing duplicates, 1136 studies were retrieved. We excluded 1044 studies through a title and abstract review because they were non-original (*n* = 236), studied other diseases (*n* = 575), were non-human studies (*n* = 11), or were written in a non-English language (*n* = 71). We reviewed 92 full-text articles. After the full-text review, 62 articles were excluded due to insufficient data (*n* = 36), lack of 2-by-2 data (*n* = 25), and not being original (*n* = 1). Finally, 30 studies [21,22,23,24,25,26,27,28,29,30,31,32,33,34,35,36,37,38,39,40,41,42,43,44,45,46,47,48,49,50] comprising 1719 patients were included in this review (Figure 1); detailed information about the eligible studies is shown in Table 1. In cases of the IJV [29,36,42], FV (was not detected), and SCV [41], we were not able to conduct the meta-analysis due to an insufficient number of studies. Two studies [36,42] reported on both the IVC and IJV. He et al. [45] reported on three subsets according to tidal volume (TV) (6 mL/kg, 9 mL/kg, 12 mL/kg) and the subset of 9 mL/kg TV showed the highest AUROC. Thus, we extracted the subset of 9 mL/kg TV. Three studies [39,40,48] reported on subsets of patients with standardized breathing and spontaneous breathing. We extracted subsets of spontaneous breathing because other studies included only patients with spontaneous breathing. Corl et al. [44] reported results obtained by both experts and novices. We extracted the results of experts because other studies were conducted by experts. One study by Blavius [50] was a comparative study between artificial intelligence and human. We extracted the result of the training dataset by humans because the number of test datasets was much smaller than the test set (20 vs. 175). Caplan et al. [48] reported different results according to the measuring site (1, 2, 3, and 5 cm apart from the aortocaval junction). We extracted a subset of 3 cm from the aortocaval junction because it was similar to other eligible studies.

### 3.2. Clinical Characteristics of Patients

All 30 eligible studies were summarized in Table 1. Twenty-eight studies [21,22,23,24,25,26,27,28,30,31,32,33,34,35,36,37,38,39,40,42,43,44,45,46,47,48,49,50] comprised the measurement of the IVC. Only three studies [29,36,42] comprised that of the IJV and one study [41] comprised that of the SCV. In our searches, there was no relevant study that comprised measurements of the FV. Twenty-three studies used the M-mode of ultrasonography [21,22,23,24,25,26,27,28,29,30,31,32,33,34,35,37,39,41,42,43,45,47,50]. Seventeen studies used echocardiography as a reference standard [21,22,24,25,26,27,30,31,32,38,39,40,45,47,48,49]. Four studies used invasive devices that extracted the waveform in arteries [29,33,34,42]. Ten studies comprised patients with sepsis [21,22,26,27,29,33,36,37,39,48] and 18 comprised those on mechanical ventilation [21,22,23,24,27,29,31,32,33,34,37,38,41,42,43,45,46,47]. In the eligible studies, the ΔIVC was measured in three ways: first, the IVC collapsibility [25,26,28,30,35,36,39,40,44,48,49,50] denotes (maximal IVC diameter—minimal IVC diameter) / IVC diameter (maximum); second, the IVC distensibility [21,23,24,27,31,32,33,34,37,38,45,46,47] denotes (maximal IVC diameter—minimal IVC diameter)/minimal IVC diameter; third, the IVC variability [22,42,43] denotes (maximal IVC diameter—minimal IVC diameter)/(minimal IVC diameter + maximal IVC diameter)/2). ΔIVC was measured near the origin of the hepatic vein via the subxiphoid view in all eligible studies. The IJV diameter was measured at the level of the cricoid cartilage. The SCV diameter was measured at the level of the clavicle.

### 3.3. DTA Review

**Figure 2:**
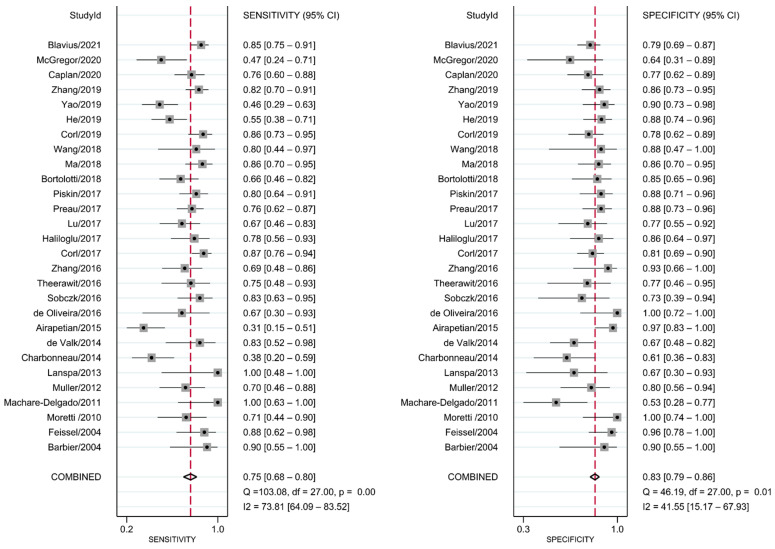
Forest plot for pooled sensitivity and specificity of respiratory variability of IVC diameter.

**Figure 3:**
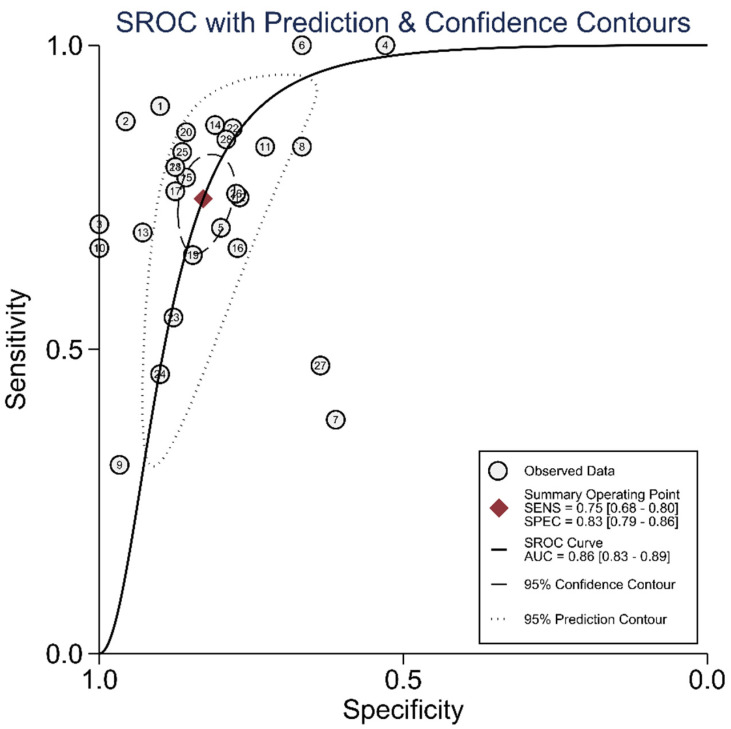
Summary ROC curve of respiratory variability of IVC diameter.

**Figure 4:**
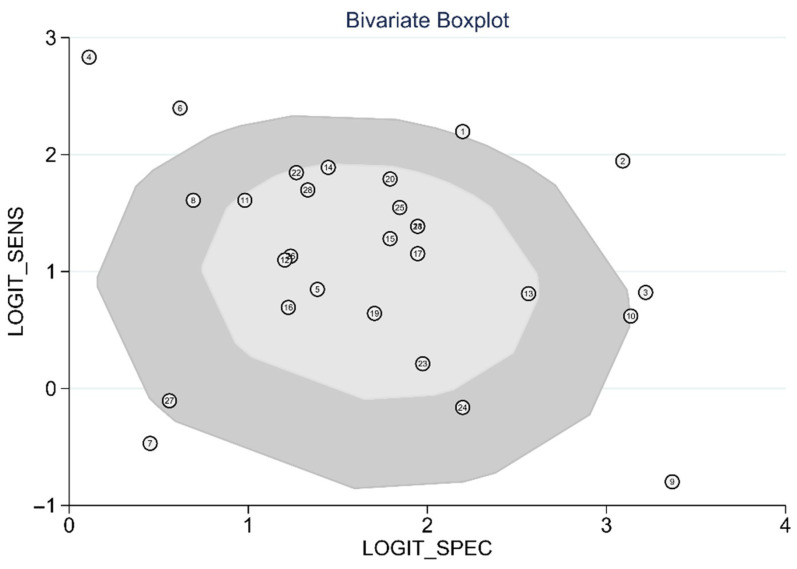
Bivariate boxplot of respiratory variability of IVC diameter.

The diagnostic test accuracy of eligible studies was summarized in Table 2. The threshold of index test (ΔIVC) ranged from 11.1 to 49%. The pooled sensitivity of ΔIVC in 28 eligible studies [21,22,23,24,25,26,27,28,30,31,32,33,34,35,36,37,38,39,40,42,43,44,45,46,47,48,49,50] was 0.75 (95% CI, 0.68–0.80, I2 = 73.8%) and the pooled specificity was 0.83 (95% CI, 0.79–0.86, I2 = 41.6%; Figure 2). The pooled positive likelihood ratio was 4.37 (95% CI, 3.58–5.33, I2 = 10.7%) and the pooled negative likelihood ratio was 0.30 (95% CI, 0.24–0.39, I2 = 75.8%). The pooled diagnostic odds ratio was 14.3 (95% CI, 10.1–20.4, I2 = 100%). The summary ROC curve (SROC) with prediction and confidence contours is depicted in Figure 3. The AUROC was 0.86 (95% CI, 0.83–0.89, I2 = 93%). To evaluate the degree of interdependence, we used a bivariate boxplot that plotted the correlation of logit-transformed sensitivity and specificity (Figure 4). Seven studies were outliers, in that these were outside the 95% confidence interval area [21,22,23,24,26,27,30]. In the test for threshold effect, Spearman’s rank correlation rho was −0.20 (*p* = 0.30). HSROC asymmetry parameter, β, was −0.676 (*p* = 0.237). Therefore, we concluded that there was no threshold effect.

### 3.4. Meta-Regression, Subgroup Analysis, and Evaluation of Heterogeneity

The univariable meta-regression and subgroup analysis using possible confounders are summarized in Table 3. We conducted the subgroup analysis according to possible confounders as follows: ΔIVC, IVC collapsibility index, reference test, ICU admission, sepsis, fluid infusion, mechanical ventilation, and the heterogeneity on a bivariate boxplot. In the meta-regression test, there was no significance of any of the moderators. There was no statistical significance in meta-regression. As in the previous meta-analysis conducted by Si et al. [13], we divided two groups who underwent MV. One group underwent MV with TV ≥ 8 mL/kg or positive end expiratory pressure (PEEP) ≤ 5 cm H_2_O [21,22,23,27,31,32,34,38,42,45]. The other group underwent MV with TV < 8 mL/kg or PEEP > 5 cm H_2_O [24,33,37,43,46]. There was no statistical significance in the meta-regression test (*p* = 0.31). We also conducted a subgroup analysis according to inliers (within 95% CI) [25,28,31,32,33,34,35,36,37,38,39,40,42,43,44,45,46,47,48,49,50] and outliers [21,22,23,24,26,27,30] on a bivariate boxplot, and the meta-regression showed no significant difference (*p* = 0.83).

### 3.5. Publication Bias

**Figure 5:**
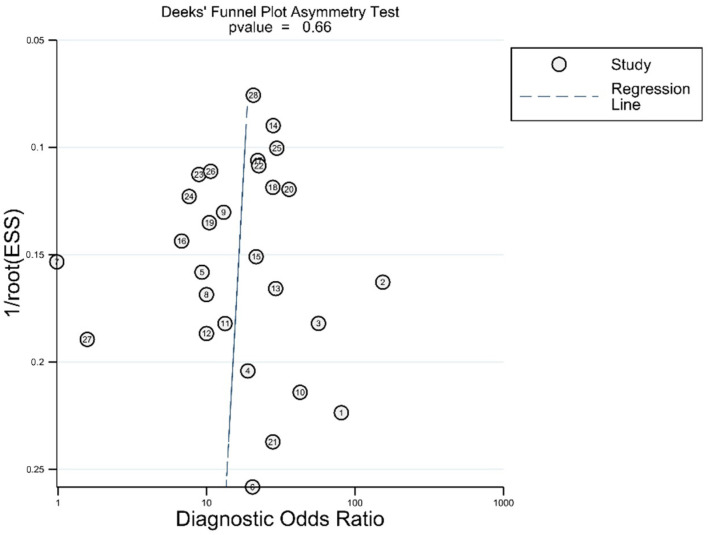
Asymmetry test for publications bias.

In Deek’s funnel plot using the diagnostic odds ratio, there was no asymmetry on visual inspection (Figure 5). There was also no statistically significant asymmetry (*p* = 0.66).

### 3.6. Quality Assessment

**Figure 6:**
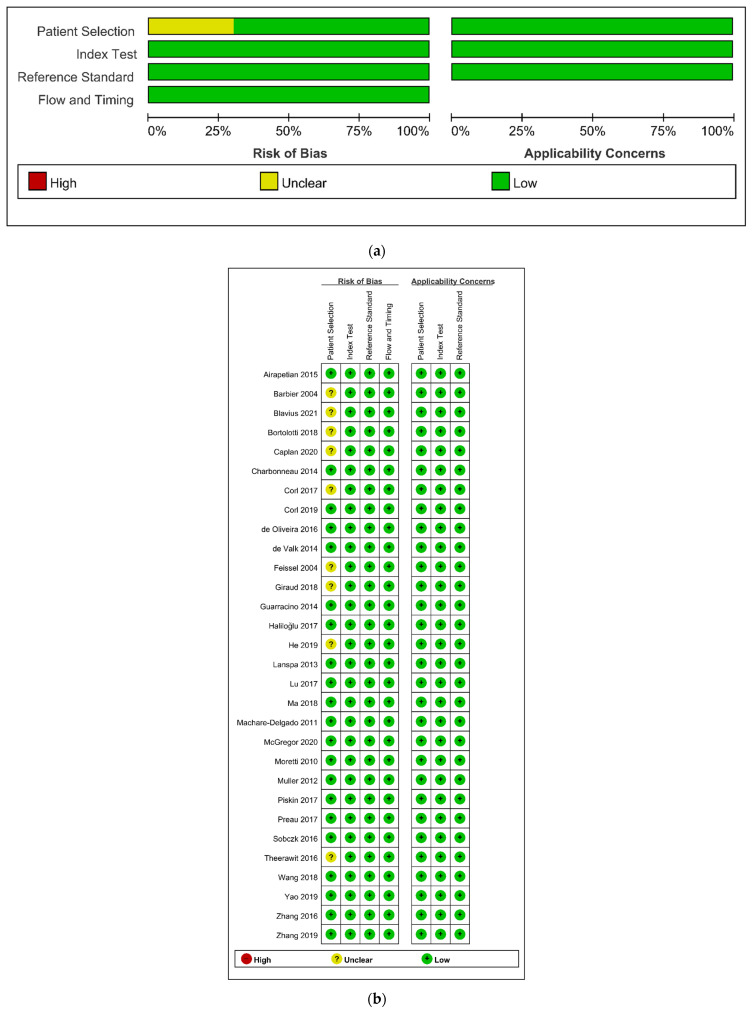
Risk of bias and applicability concerns graph (**a**) and summary (**b**): review authors’ judgements about each domain presented as percentages across included studies.

The details of the quality assessment are depicted in Figure 6. In terms of patient selection, the risk of bias was unclear in nine studies (30.0%) [21,22,33,35,40,41,45,48,50]. Consequently, these studies showed no consecutive patient selection or no description of it. In other domains of QUDAS-2 assessment, all studies showed a low risk of bias.

## 4. Discussion

Our results suggest that the diagnostic accuracy of ultrasonographic ΔIVC for predicting fluid responsiveness is acceptable. The pooled sensitivity, specificity, positive likelihood ratio, negative likelihood ratio, diagnostic odds ratio, and AUROC of ΔIVC were 0.75, 0.83, 4.37, 0.30, 14.3, and 0.86, respectively. In the subgroup analysis, there was no difference between patients on MV and those breathing spontaneously. Despite the systematic review, we found only three studies on the IJV and one on the FV. We found no study on the SCV. There was insufficient evidence to support the diametric measurement of these large veins as an alternative to that of the IVC. More prospective studies are warranted, which should consider the threshold of the index test and the heterogeneity of the reference standard.

Recently, several previous systematic reviews and meta-analyses were conducted to investigate the diagnostic accuracy of ΔIVC. Orso et al. [12], in a meta-analysis including 20 studies with ΔIVC, reported that the pooled sensitivity, specificity, and AUROC were 0.71, 0.75, and 0.71, respectively. They included several studies of pediatric patients, whereas we excluded these studies. Si et al. [13], in a meta-analysis including 12 studies comprising only patients on MV, reported a sensitivity, specificity, and AUROC of 0.73, 0.82, and 0.85, respectively. In our subgroup analysis, studies comprising patients on MV showed a sensitivity, specificity, and AUROC of 0.74, 0.85, and 0.87, respectively, whereas studies comprising patients with spontaneous breathing showed similar results, with a sensitivity, specificity, and AUROC of 0.75, 0.81, and 0.85, respectively. Si et al. [13] concluded that ΔIVC was a poor predictor in patients with TV < 8 mL/kg or PEEP > 5 cm H_2_O through subgroup analysis (k = 6) (sensitivity, specificity, and AUROC of 0.66, 0.68, and 0.70, respectively). However, in our subgroup analysis (k = 5), ΔIVC in this setting showed better results, which were a sensitivity, specificity, and AUROC of 0.73, 0.77, and 0.82, respectively. In our analysis, similar to that of Si et al. [13], the performance of ΔIVC was higher in patients with TV ≥ 8 mL/kg or PEEP ≤ 5 cm H_2_O (sensitivity, specificity, and AUROC of 0.74, 0.88, and 0.90), but the meta-regression test did not show a significant difference (*p* = 0.31). Overall, compared with previous meta-analyses [12,13], we updated our interpretation with data from 11 studies that have been published since 2018. However, two studies [51,52] in the previous meta-analyses by Orso et al. [12] and one study [53] in the other meta-analysis by Si et al. [13] did not have 2-by-2 contingency data in our recalculation. Thus, we excluded these three studies. Only one previous meta-analysis investigated the IVC diameter, without a delineation of respiratory variation [54]. They analyzed two case–control and three before-and-after studies. They found a significantly lower diameter of the IVC in hypovolemic status and the mean difference was 6.3 mm (95% CI, 6.0–6.5). However, this effect size is apparently too small to use in clinical practice. Indeed, the inherent size of the IVC may vary in each patient. Similar static index tests, such as central venous pressure, showed no clinical significance in the previous study [55]. Since this study was published, there has been no meta-analysis investigating the IVC diameter alone. In common with ΔIVC, a more dynamic index would be appropriate for evaluating volume status.

Due to the limited number of studies that met our inclusion criteria, we did not conduct the meta-analysis for the IJV, SCV, and FV. We found only three studies that evaluated the IJV [29,36,42]. The specificity, sensitivity, and AUROC of these studies were sufficiently high for predicting fluid responsiveness. The AUROC of the IJV ranged from 0.825 to 0.915. The AUROC of the SCV was also sufficient, with a value of 0.970. Both the IJV and the SCV are located in proximity of the right atrium. Thus, these would be alternative vessels to investigate. However, the FV would be limited due to its distance from the right atrium. One eligible study in our meta-analysis reported a strong correlation between IVC-CI and IJV-CI (r = 0.976, *n* = 44) [36]. One study that was excluded because there was no fluid challenge, reporting a moderately strong correlation between IVC-CI and SCV-CI (r = 0.781, *n* = 34) [56]. In the case of the FV, only one study was excluded because it reported only a modest correlation between IVC-CI and FV-CI (r = 0.642, 57) [57]. In future reviews, the IJV and SCV need to be further investigated.

In the eligible studies of our meta-analysis, several conventional reference standards were used after fluid loading to determine the fluid responsiveness. The increase in CO or SV was considered as a response to fluid replacement. Therefore, the accurate measurement of CO or SV is crucial. To measure CO or SV, the most reliable method is the insertion of a Swan–Ganz catheter [4]. This involves an injection of ice-cold water into the right atrium through a pulmonary artery catheter and measurement of CO or SV using the temperature change [58]. It measures SvO2 to reflect accurate, real-time change in hemodynamics [59]. However, it is a difficult technique to perform in practice, especially if indicated often, and has limitations because it is invasive and even more difficult to perform in the presence of arrhythmias, pulmonary infarction, or catheter injury with vascular complications [60]. In our analysis, no study used a Swan–Ganz catheter as a reference standard. Another way to measure CO or SV is to extract the arterial waveform. Since the SV is estimated using the area under the dicrotic notch at the start of the rise of arterial pressure, the SV can be calculated for every heartbeat [59,61]. VigileoTM (Edwards life science, Irvine, CA, USA), MostCare™ (Vytech, Padova, Italy) using PRAM (Pressure Recording Analytical Method), and PiCCO^®^ (Pulsion Medical Systems, Munich, Germany), which uses blood pressure waveforms, were proposed as less invasive methods [60,61]. These involve the insertion of a central venous catheter and a relatively small-sized device, approximately 4–5 Fr, into the artery, and allow the monitoring of continuous values even when the patient is unstable. The arterial waveform analysis method is less invasive than the Swan–Ganz method. However, re-calibration is required every 6–12 h in the case of vascular elasticity, aortic insufficiency, or inaccurate arterial pressure waveforms [60,61]. The method of measuring CO or SV using echocardiography involves measuring the velocity-time integral using the diameter of the left ventricular outlet and Doppler ultrasound [62]. Echocardiography is useful because it can also provide the differential diagnosis of cardiac dysfunction and hypovolemia by measuring chamber size and cardiac function. However, it is not able to detect continuous changes like the Swan–Ganz catheter and should be performed by an expert who has a high level of experience in general [63]. The bioimpedance method can measure the CO or SV only by direct contact [64]. The fluctuation of the volume of the body with pulsatile changes results in electrical impedance, and the variation of the systolic period is measured, allowing the value of the CO or SV to be monitored [65]. However, reliability is limited in some critically ill patients, and appropriate improvements are likely to be necessary in future studies. Evidence for the superiority of one method over another from the above techniques is limited [10]. We assumed that these reference standards have similar diagnostic accuracy.

Our analysis has several limitations. First, all eligible studies were observational. Second, several eligible studies have an unclear risk of bias in terms of patient selection. Third, the threshold of the index test varied and there was considerable heterogeneity. To overcome this issue, we investigated the correlation between sensitivity and specificity to detect the threshold effect. Fourth, the reference standard was heterogeneous. We also conducted a meta-regression test to evaluate the heterogeneity. Fifth, both patients on MV and those breathing spontaneously were included, although the physiology of the two is antonymous. We conducted a meta-regression, which showed no significance. Sixth, we did not find sufficient eligible studies involving the IJV and SCV. We found only three studies on the IJV. We did not conduct the meta-analysis due to statistical instability. We found no study that measured the respiratory variation of the FV diameter. Future studies are needed to investigate and correct the above deficiencies. Seventh, there would exist a “grey zone” to discriminate response to fluid resuscitation even though the ΔIVC is an easy-to-determine quantitative variable. Thus, integrating an additional qualitative sonographic evaluation may be more helpful in future study [66]. Finally, we included only published original articles and those written in English. This would be expected to introduce publication bias; however, this was not noted in our analysis.

## 5. Conclusions

Our systematic review and meta-analysis suggest that the ultrasonographic measurement of the respiratory variation in the diameter of the IVC has a favorable diagnostic accuracy for predicting fluid responsiveness in critically ill patients. However, we concluded that there is insufficient evidence in the case of the IJV, SCV, and FV diameters to have clinical application.

## Figures and Tables

**Table 1:** Main characteristics of the eligible studies.

Author	Year	Target Vein	Index Test	Device (Index Test)	Measure Site	Reference Standard	Device (Reference Standard)	Setting	Threshold (Reference Standard)	Fluid Challenge	MV	MV Setting	Study Design	Location	Study Period
Barbier [21]	2004	IVC	IVC-d	US, M-mode	Just upstream of the origin of hepatic vein	CI	ECHO	ICU, adult, MV, septic shock	CI = 15%	7ml/kg 4% modified fluid gelatin over 30 min	yes	Tidal = 8.5 ± 1.5 mL/kg, PEEP = 4 ± 2 cm H_2_O	Obs	France	January 2003–July 2003
Feissel [22]	2004	IVC	IVC-v	US, M-mode	Approximately 3 cm from the RA	CO	ECHO	ICU, adult, MV, septic shock	CO = 15%	8 mL/kg of 6% hydroxyethyl starch over 20 min	yes	Tidal = 8 to 10 mL/kg	Obs	USA	NR
Moretti [23]	2010	IVC	IVC-d	US, M-mode	2 cm upstream of the origin of hepatic vein	SVV	transpulmonary thermodilution	ICU, adult, MV, SAH	CI = 15%	7 mL/kg of 6% hydroxyethyl starch over 30 min	yes	Tidal = 8 mL/kg, PEEP = 5 cm H_2_O	Obs	Italy	August 2008–July 2009
Machare-Delgado [24]	2011	IVC	IVC-d	US, M-mode	2 cm upstream of the origin of hepatic vein	SVI	ECHO	ICU, vasopressor dependent with MV	SVI = 10%	500 mL NS over 10 min	yes	Tidal = 8 mL/kg, PEEP = 6.8 ± 2.8 cm H_2_O	Obs	USA	NR
Muller [25]	2012	IVC	IVC-c	US, M-mode	2–3 cm upstream of the origin of hepatic vein	VTI	ECHO	ICU, adult, spontaneous breathing with ACF	subaortic VTI = 10%	500 mL of 6% hydroxyethyl starch over 15 min	no		Obs	France	April 2009–April 2011
Lanspa [26]	2013	IVC	IVC-c	US, M-mode	0.5–3 cm upstream of the origin of hepatic vein	CI	ECHO	ICU, adult, septic shock, spontaneous breathing	CI = 15%	10 mL/kg of crystalloid over a period of less than 20 min	no		Obs	USA	January 2010–April 2011
Charbonneau [27]	2014	IVC	IVC-d	US, M-mode	Just upstream of the origin of hepatic vein	CI	ECHO	ICU, adult, septic shock with MV	CO = 15%	7 mL/kg of 6% hydroxyethyl starch over 15 min	yes	Tidal = 8 to 10 mL/kg	Obs	France	NR
de Valk [28]	2014	IVC	IVC-c	US, M-mode	3 cm upstream of the origin of hepatic vein	SBP	Non-invasive BP measurement	ED, adult, spontaneous breathing with sign of shock	SBP = 10mmHg	500 mL NS over 15 min	no		Obs	Netherlands	NR
Guarracino [29]	2014	IJV	IJV-d	US, M-mode	At the cricoid cartilage level	CI	PRAM	ICU, adult, sepsis, MV	CI = 15%	7 mL/kg of crystalloid over 30 min	yes	Tidal = 6 to 8 mL/kg, PEEP = 6 cm H_2_O	Obs	USA	October 2012–December 2013
Airapetian [30]	2015	IVC	IVC-c	US, M-mode	2 cm upstream of the origin of hepatic vein	CO	ECHO	ICU, adult, spontaneous breathing decided to perform volume expansion	CO = 10%	PLR, 500 mL NS over 15 min	no		Obs	France	NR
de Oliveira [31]	2016	IVC	IVC-d	US, M-mode	Just upstream of the origin of hepatic vein	VTI	ECHO	ICU, adult, MV, immediate perioperative period within the first 24 h	VTI = 15%	500 mL NS over 15 min	yes	Tidal = 8 mL/kg	Obs	Brazil	February 2013–September 2014
Sobczk [32]	2016	IVC	IVC-d	US, M-mode	Just upstream of the origin of hepatic vein	CO	ECHO	ICU, adult, coronary bypass grafting, MV	CO = 15%	250 mL NS	yes	Tidal = 8 mL/kg, PEEP = 4.5 cm H_2_O	Obs	Poland	NR
Theerawit [33]	2016	IVC	IVC-d	US, M-mode	2 cm upstream of the origin of hepatic vein	CO	Vigileo/FloTrac monitor	ICU, adult, septic shock	CO = 15%	1000 mL of crystalloid over 1 h or 500 mL of colloid (6% hydroxyethyl starch or 5% human albumin) over 30 min	yes	Tidal ≥ 8 mL/kg, PEEP = 8 to 10 cm H_2_O	Obs	Thailand	November 2012–December 2013
Zhang [34]	2016	IVC	IVC-d	US, M-mode	2 cm upstream of the origin of hepatic vein	SVI	Vigileo/FloTrac monitor	OR, adult, Anesthesia for gastrointestinal surgery, ASA I or II	SVI = 15%	7 mL/kg of 6% hydroxyethyl starch over 30 min	yes	Tidal = 8 to 10 mL/kg	Obs	China	NR
Corl [35]	2017	IVC	IVC-c	US, M-mode	Just upstream of the origin of hepatic vein	CI	NICOM	ICU, adult, spontaneous breathing, acute circulatory failure	CI = 10%	3 min PLR, 500 mL NS bolus	no		Obs	USA, two hospitals	August 2014–July 2016
Haliloğlu [36]	2017	IVC	IVC-c	US, B-mode	IVC—0.5 to 3cm upstream of the origin of hepatic vein	CI	USCOM	ICU, adult, sepsis, spontaneous breathing	CI = 15%	PLR	no		Obs	Turkey	NR
		IJV	IJV-c	US, B-mode	IJV—at the cricoid cartilage level	CI	USCOM	ICU, adult, sepsis, spontaneous breathing	CI = 15%	PLR	no		Obs	Turkey	NR
Lu [37]	2017	IVC	IVC-d	US, M-mode	2 cm upstream of the origin of hepatic vein	CI	PiCCO	ICU, adult, septic shock, MV	CI = 10%	200 mL NS over 10 min	yes	Tidal = 8 to 10 mL/kg, PEEP = 5 to 12 cm H_2_O	Obs	China	January 2012–December 2015
Piskin [38]	2017	IVC	IVC-d	US	NR	CI	ECHO	ICU, adult, MV	CI = 15%	PLR	yes	Tidal = 8 mL/kg	Obs	Turkey	April 2016–November 2016
Preau [39]	2017	IVC	IVC-c	US, M-mode	1.5 to 2 cm upstream of the origin of hepatic vein	SVI	ECHO	ICU, adult, spontaneous breathing, sepsis, acute circulatory failure	SVI = 10%	500 mL of 4% gelatin over 30 min	no		Obs	France, two hospitals	November 2011–January 2014
Bortolotti [40]	2018	IVC	IVC-c	US	1.5 to 2 cm upstream of the origin of hepatic vein	VTI	ECHO	ICU, adult, spontaneous breathing, infection, acute circulatory failure, cardiac arrythmia	VTI = 10%	500 mL of 4% gelatin over 30 min	no		Obs	France, two hospitals	May 2012–May 2015
Giraud [41]	2018	SCV	SCV-c	US, M-mode	Clavicle	CO	PiCCO	ICU, adult, MV	CO = 15%	500 mL NS over 10 min	yes	NR	Obs	Swiss	2009–2010
Ma [42]	2018	IVC	IVC-v	US, M-mode	2cm from right atrium	SV	Vigileo/FloTrac monitor	ICU, adult who underwent cardiac surgery, circulatory instability	SV = 15%	PLR, 500 mL Gelofusine over 300 min	yes	Tidal = 8 mL/kg, PEEP = 5 cm H_2_O	Obs	China	August 2016–December 2016
		IJV	IJV-v	US, M-mode	At the cricoid cartilage level	SV	Vigileo/FloTrac monitor	ICU, adult who underwent cardiac surgery, circulatory instability	SV = 15%	PLR, 500 mL Gelofusine over 300 min	yes	Tidal = 8 mL/kg, PEEP = 5 cm H_2_O	Obs	China	August 2016–Dec ember 2016
Wang [43]	2018	IVC	IVC-v	US, M-mode	2 cm from right atrium	CI	PiCCO	ICU, adult, MV, postpneumonectomy, requiring fluid resuscitation	CI = 15%	7 mL/kg of 6% hydroxyethyl starch over 30 min	yes	Tidal = 8 to 12 mL/kg, PEEP = 5 to 10 cm H_2_O	Obs	China	August 2014–December 2016
Corl [44]	2019	IVC	IVC-c	US, B-mode	3 cm from right atrium	CI	NICOM	ICU, adult, acute circulatory failure, spontaneous breathing	CI = 10%	500 mL NS bolus	no		Obs	USA	November 2016–July 2018
He [45]	2019	IVC	IVC-d	US, M-mode	2 to 3 cm from right atrium	VTI	ECHO	OR, adult, general anesthesia, elective surgery mechanical ventilation	VTI = 15%	6 mL/kg of 4% gelatin over 10 min	yes	Tidal = 6, 9, 12 mL/kg	Obs	China	June 2018–September 2018
Yao [46]	2019	IVC	IVC-d	US, B-mode	2 to 3 cm from right atrium	CO	CNAP	ICU, adult, MV	CO = 10%	PLR	yes	Tidal = 7.6 mL/kg (responder), 7.8 mL/kg (non-responder); PEEP = 5 cm H_2_O	Obs	China	December 2017–March 2018
Zhang [47]	2019	IVC	IVC-d	US, M-mode	Just upstream of the origin of the supraheptic vein	VTI	ECHO	ICU, adult, MV, need of an assessment of fluid responsiveness	VTI = 10%	PLR	yes	NR	Obs	China	July 2018–January 2019
Caplan [48]	2020	IVC	IVC-c	US, B-mode	1, 3, 4, 5 cm from aortocaval junction	SVI	ECHO	ICU, adult, sepsis, acute circulatory failure, two cohort (normal sinus rhythm and arrythmia), spontaneous breathing	SVI = 10%	500 mL of 4% gelatin over 30 min	no		Obs	France	November 2011–May 2015
McGregor [49]	2020	IVC	IVC-c	US, B-mode	2 to 3 cm from right atrium	VTI	ECHO	ED, adult, IV fluid required, spontaneous breathing	VTI = 10%	250–500 mL NS over 15 min or less	no		Obs	UK	NR
Blavius [50]	2021	IVC	IVC-c	US	NR	CI	Non-invasive cardiac output monitoring	ICU, adult, critically ill patients, spontaneous breathing	CI = 10%	500 mL NS bolus	no		Obs	USA	NR

IVC, inferior vena cava; IJV, internal jugular vein; SCV, subclavian vein; IVC-d, IVC distensibility; IVC-c, IVC collapsibility; IVC-v, IVC variability; IJV-d, IJV distensibility, IJV-c, collapsibility; IJV-v, IJV variability; SCV-c, SCV collapsibility; ECHO, echocardiography; PRAM, pressure recording analytical method; NICOM, non-invasive cardiac output monitoring; PiCCO, pulse index continuous cardiac output; USCOM, ultrasonic cardiac output monitor; CNAP, continuously monitored by continuous non-invasive arterial pressure; PLR, passive leg raising; US, ultrasound; SVV, stroke volume variation; SVI, stroke volume index; SBP, systolic blood pressure; VTI, velocity time integral; CO, cardiac output; CI, cardiac index; ICU, intensive care unit; ED, emergency department; OR, operating room; MV, mechanical ventilation; PEEP, positive end expiratory pressure; SAH, subarachnoid hemorrhage; Obs, observational study; NR, not reported.

**Table 2:** Diagnostic test accuracy of eligible studies.

Author	Year	Target Vein	TP	FP	FN	TN	Sen	Spe	AUROC	*n*	Threshold (Index Test)
Barbier [21]	2004	IVC	9	1	1	9	0.90	0.90	0.910	20	ΔIVC = 18%
Feissel [22]	2004	IVC	14	1	2	22	0.88	0.96	NR	39	ΔIVC = 12%
Moretti [23]	2010	IVC	12	0	5	12	0.71	1.00	0.902	29	ΔIVC = 16%
Machare-Delgado [24]	2011	IVC	8	8	0	9	1.00	0.53	0.816	25	ΔIVC = 12%
Muller [25]	2012	IVC	14	4	6	16	0.70	0.80	0.770	40	ΔIVC = 40%
Lanspa [26]	2013	IVC	5	3	0	6	1.00	0.67	0.840	14	ΔIVC = 15%
Charbonneau [27]	2014	IVC	10	7	16	11	0.38	0.61	0.430	44	ΔIVC = 21%
de Valk [28]	2014	IVC	10	11	2	22	0.83	0.67	0.741	45	ΔIVC = 36.5%
Guarracino [29]	2014	IJV	24	1	6	19	0.80	0.95	0.915	50	ΔIJV = 18%
Airapetian [30]	2015	IVC	9	1	20	29	0.31	0.97	0.620	59	ΔIVC = 49%
de Oliveira [31]	2016	IVC	6	0	3	11	0.67	1.00	0.840	20	ΔIVC = 16%
Sobczk [32]	2016	IVC	20	3	4	8	0.83	0.73	0.739	35	ΔIVC = 18%
Theerawit [33]	2016	IVC	12	3	4	10	0.75	0.77	0.688	29	ΔIVC = 10.7%
Zhang [34]	2016	IVC	18	1	8	13	0.69	0.93	0.850	40	ΔIVC = 46%
Corl [35]	2017	IVC	53	12	8	51	0.87	0.81	0.840	124	ΔIVC = 25%
Haliloğlu [36]	2017	IVC	18	3	5	18	0.78	0.86	0.825	44	ΔIVC = 35%
		IJV	18	3	5	18	0.78	0.86	0.825	44	ΔIJV = 36%
Lu [37]	2017	IVC	18	5	9	17	0.67	0.77	0.805	49	ΔIVC = 20%
Piskin [38]	2017	IVC	32	4	8	28	0.80	0.88	0.928	72	ΔIVC = 23.08%
Preau [39]	2017	IVC	38	5	12	35	0.76	0.88	0.820	90	ΔIVC = 31%
Bortolotti [40]	2018	IVC	19	4	10	22	0.66	0.85	0.820	55	ΔIVC = 37%
Giraud [41]	2018	SCV	9	1	0	10	1.00	0.91	0.970	20	ΔSVC = 13.4%
Ma [42]	2018	IVC	30	5	5	30	0.86	0.86	0.830	70	ΔIVC = 13.39%
		IJV	32	6	3	29	0.91	0.83	0.880	70	ΔIJV = 12.99%
Wang [43]	2018	IVC	8	1	2	7	0.80	0.88	0.860	18	ΔIVC = 15%
Corl [44]	2019	IVC–expert	38	9	6	32	0.86	0.78	0.820	85	ΔIVC = 25%
		IVC-novice	35	13	9	28	0.70	0.68	0.690	85	ΔIVC = 25%
He [45]	2019	IVC-tidal 6mL/kg	26	10	12	31	0.68	0.76	0.710	79	ΔIVC = 11.1%
		IVC-tidal 9mL/kg	21	5	17	36	0.55	0.88	0.790	79	ΔIVC = 15.3%
		IVC-tidal 12mL/kg	20	5	18	36	0.53	0.88	0.730	79	ΔIVC = 13.4%
Yao [46]	2019	IVC	17	3	20	27	0.46	0.90	0.702	67	ΔIVC = 25.6%
Zhang [47]	2019	IVC	47	6	10	38	0.82	0.86	0.815	101	ΔIVC = 14.5%
Caplan [48]	2020	IVC	31	9	10	31	0.76	0.77	0.820	81	ΔIVC = 20%
McGregor [49]	2020	IVC	9	4	10	7	0.47	0.64	0.464	30	ΔIVC = 40%
Blavius [50]	2021	IVC—training set	71	19	13	72	0.85	0.79	0.820	175	ΔIVC = 25%
		IVC—test set	8	0	1	11	0.89	1.00	0.940	20	ΔIVC = 25%

IVC, inferior vena cava; IJV, internal jugular vein; SCV, subclavian vein; TP, true positive; FP, false positive; FN, false negative; TN, true negative; Sen, sensitivity; Spe, specificity; AUROC, area under receiver operator characteristic curve; ΔIVC, respiratory variation of inferior vena cava diameter; ΔIJV, respiratory variation of internal jugular vein diameter; ΔSCV, respiratory variation of subclavian vein diameter.

**Table 3:** Meta-regression and subgroup analysis of respiratory variability of IVC diameter.

Subgroup by Moderator	Pooled Sen (95% CI)	Pooled Spe (95% CI)	Pooled PLR (95% CI)	Pooled NLR (95% CI)	Pooled DOR (95% CI)	AUROC (95% CI)	Meta -Regression Test (*p* Value)
ΔIVC ≥ 20%							0.07
yes (k = 16) [25,27,28,30,34,35,36,37,38,39,40,44,46,48,49,50]	0.70 (0.61–0.78)	0.81 (0.78–0.85)	3.86 (3.12–4.76)	0.36 (0.27–0.48)	10 (7–16)	0.84 (0.80–0.87)	
no (k = 12) [21,22,23,24,26,31,32,33,42,43,45,47]	0.81 (0.72–0.88)	0.85 (0.77–0.91)	5.42 (3.61–8.14)	0.22 (0.15–0.33)	25 (14–42)	0.90 (0.87–0.92)	
Using IVC collapsibility index							0.58
yes (k = 12) [25,26,28,30,35,36,39,40,44,48,49,50]	0.76 (0.65–0.84)	0.81 (0.76–0.86)	4.10 (3.30–5.10)	0.30 (0.20–0.43)	14 (9–22)	0.85 (0.82–0.88)	
no (k = 16) [21,22,23,24,27,31,32,33,34,37,38,42,43,45,46,47]	0.74 (0.65–0.81)	0.85 (0.79–0.90)	5.06 (3.36–7.55)	0.31 (0.22–0.42)	17 (9–31)	0.87 (0.84–0.90)	
Using echocardiography as a reference test							0.68
yes (k= 17) [21,22,24,25,26,27,30,31,32,36,38,39,40,45,47,48,49]	0.73 (0.63–0.81)	0.83 (0.77–0.88)	4.39 (3.13–6.14)	0.33 (0.24–0.46)	13 (8–24)	0.86 (0.83–0.89)	
no (k = 11) [23,28,33,34,35,37,42,43,44,46,50]	0.78 (0.69–0.85)	0.82 (0.77–0.87)	4.40 (3.40–5.80)	0.27 (0.19–0.37)	16 (10–26)	0.87 (0.84–0.90)	
ICU patient							0.22
yes (k = 24) [21,22,23,24,25,26,27,29,30,31,32,33,35,36,37,38,39,40,41,42,43,44,46,47,48,50]	0.76 (0.69–0.82)	0.83 (0.80–0.86)	4.60 (3.80–5.50)	0.28 (0.22–0.37)	16 (11–23)	0.87 (0.83–0.89)	
no (ED, OR) (k = 4) [28,34,45,49]	0.62 (0.50–0.73)	0.80 (0.66–0.89)	3.00 (1.70–5.30)	0.48 (0.35–0.66)	6 (3–14)	0.70 (0.66–0.74)	
Sepsis patients							0.81
yes (k = 9) [21,22,26,27,33,36,37,39,48]	0.75 (0.63–0.83)	0.82 (0.73–0.88)	4.13 (2.52–6.76)	0.31 (0.20–0.49)	13 (5–33)	0.87 (0.83–0.92)	
no (k = 19) [23,24,25,28,29,30,31,32,34,35,38,40,41,42,43,44,45,46,47,49,50]	0.75 (0.66–0.82)	0.84 (0.79–0.88)	4.77 (3.77–6.03)	0.30 (0.22–0.40)	16 (11–23)	0.88 (0.84–0.90)	
Fluid challenge							0.34
PLR only (k = 4) [36,38,46,47]	0.73 (0.57–0.85)	0.88 (0.80–0.92)	5.90 (3.60–9.50)	0.30 (0.18–0.52)	19 (8–44)	0.89 (0.86–0.91)	
Fluid infusion (k = 24) [21,22,23,24,25,26,27,28,29,30,31,32,33,34,35,37,39,40,41,42,43,44,45,48,49,50]	0.75 (0.68–0.81)	0.82 (0.77–0.86)	4.15 (3.31–5.22)	0.31 (0.23–0.40)	14 (9–20)	0.86 (0.83–0.89)	
Mechanical Ventilation							0.58
yes (k16) [21,22,23,24,27,31,32,33,34,37,38,42,43,45,46,47]	0.74 (0.65–0.81)	0.85 (0.79–0.90)	5.06 (3.39–7.54)	0.31 (0.22–0.42)	17 (9–31)	0.87 (0.848–0.90)	
no (k = 12) [25,26,28,30,35,36,39,40,44,48,49,50]	0.76 (0.65–0.84)	0.81 (0.76–0.86)	4.10 (3.30–5.10)	0.30 (0.20–0.43)	14 (9–22)	0.85 (0.82–0.88)	
Mechanical Ventilation Setting (k = 15) [21,22,23,24,27,31,32,33,34,37,38,42,43,45,46]							0.31
Tidal < 8mL/kg or PEEP > 5 mmHg (k = 5) [24,33,37,43,46]	0.73 (0.52–0.87)	0.77 (0.62–0.88)	3.24 (1.97–5.3)	0.35 (0.19–0.64)	9 (4–21)	0.82 (0.35–0.97)	
Tidal ≥ 8mL/kg or PEEP ≤ 5 mmHg (k = 10) [21,22,23,27,31,32,34,38,42,45]	0.74 (0.63–0.83	0.88 (0.81–0.93)	6.19 (3.55–10.80)	0.29 (0.19–0.45)	21 (9–52)	0.90 (0.17–1.00)	
Bivariate boxplot							0.83
inlier (k = 21) [21,22,23,24,26,27,30]	0.75 (0.70–0.80)	0.83 (0.79–0.86)	4.32 (3.60–5.20)	0.30 (0.24–0.37)	14 (10–20)	0.86 (0.42–0.98)	
outlier (k = 7) [25,28,29,31,32,33,34,35,36,37,38,39,40,41,42,43,44,45,46,47,48,49,50]	0.80 (0.48–0.95)	0.87 (0.66–0.96)	6.02 (2.20–16.42)	0.23 (0.07–0.73)	27 (5–142)	0.91 (0.17–1.00)	

ΔIVC, respiratory variation of inferior vena cava; IVC, inferior vena cava; ICU, intensive care unit; ED, emergency department; OR, operating room; PLR, passive leg raising test; PEEP, positive end-expiratory pressure; CI, confidence interval; Sen, sensitivity; Spe, specificity; PLR, positive likelihood ratio; NLR, negative likelihood ratio; DOR, diagnostic odds ratio; AUROC, area under receiver operating characteristic curve.

## Data Availability

Not applicable.
